# A comprehensive evaluation of factors affecting nurse leaders’ work-related well-being

**DOI:** 10.1108/LHS-12-2021-0098

**Published:** 2022-05-13

**Authors:** Milja Niinihuhta, Anja Terkamo-Moisio, Tarja Kvist, Arja Häggman-Laitila

**Affiliations:** Department of Nursing Science, University of Eastern Finland, Kuopio, Finland

**Keywords:** Nurse leader, Nurse manager, Nurse director, Work-related well-being, Well-being at work

## Abstract

**Purpose:**

This study aims to describe nurse leaders’ experiences of work-related well-being and its association with background variables, working conditions, work engagement, sense of coherence and burnout.

**Design/methodology/approach:**

An electronic survey design was used. Data was collected between December 2015 and May 2016 with an instrument that included demographic questions and four internationally validated scales: the Utrecht Work Engagement Scale, QPS Nordic 34+, the shortened Sense of Coherence scale and the Maslach Burnout Inventory. Data was analysed using statistical methods.

**Findings:**

A total of 155 nurse leaders completed the questionnaire, giving a 44% response rate. Most of them worked as nurse managers (89%). Participants’ work-related well-being scores ranged from 8 to 10. Statistically significant relationships were found between participants’ work-related well-being and their leadership skills, current position, sense of coherence and levels of burnout. In addition, there were statistically significant relationships between work-related well-being and all dimensions of working conditions.

**Originality/value:**

This study underlines the fact that work-related well-being should not be evaluated based on a single factor. The participants’ perceived work-related well-being was high, although almost half of them reported always or often experiencing stress. The results suggest that nurse leaders may have resources such as good leadership and problem-solving skills, supportive working conditions and a high sense of coherence that prevent the experienced stress from adversely affecting their work-related well-being.

## Introduction

Work-related well-being can be conceptualized in many ways, depending on factors such as the field in which the studied employees work, the organization that they work for and the country in which they work. In addition, it is a very complex concept that encompasses social, physical, psychological and emotional factors both inside and outside the workplace. As a result, some conceptualizations of work-related well-being focus only on employees’ mental health experiences while others also include work-related and personal characteristics (Buffet *et al.*, 2013). Trinchero *et al.* (2014) defined three categories of work-related well-being, namely, physical, social and psychological well-being. Moreover, they state that high supervisor support results in high levels of work-related well-being. Work-related well-being can be described as a variety of emotional experiences and attitudes at work and their influence on personal and organization-related outcomes (Brunetto *et al.*, 2013; Sudha *et al.*, 2016). In this study, work-related well-being is understood as a comprehensive concept that includes employees’ experiences of their well-being, resources, state of burnout and perceptions of their work conditions and professional capabilities (Buffet *et al.*, 2013).

Nurse leaders play a pivotal role in health-care organizations (Remegio *et al.*, 2020; Saleh *et al.*, 2018; Adriaenssens *et al.*, 2017), and the demands placed upon them by organizations, nursing staff and patients have increased in recent decades. In addition, their role has changed; they are increasingly expected to actively coach staff and involve staff in decision-making. Nurse leaders are also expected to lead their employees towards outstanding and high-quality and effective care rather than simply being outstanding clinicians themselves (Nurmeksela *et al.*, 2020; Siirala, 2020; Adams *et al.*, 2019). Turnover rates of nurse leaders are estimated to be between 5.8% and 25%, depending on the country, and are expected to increase as the baby boomer generation of nurse leaders retires (Labrague, 2020). At the same time, the recruitment of new nurse leaders has become more challenging, and there are growing concerns about stress and burnout among nurse managers and the appeal of a career as a nurse manager (Kelly *et al.*, 2019; Saifman and Sherman, 2019; Djukic *et al.*, 2017). It is therefore essential for health-care organizations to learn how to effectively recruit and retain new nurse managers (Djukic *et al.*, 2017).

Job stress and work–life conflicts are often mentioned as important indicators of turnover intentions in nursing staff and are likely to be important for turnover among nurse leaders as well (Labrague *et al.*, 2018). Job stress can be defined as harmful physical and emotional responses that occur when there is imbalance between the requirements of the job and the capabilities, resources or needs of the worker (Abram and Jacobowitz, 2021). It may be seen resulting from contradiction between the work-related demands and the coping skills of an individual (Labrague, 2020). As Maslach and Leiter (2016) pointed out, stress is inherent in health care, and also in leadership. Although work in the health-care sector can be rewarding (Maslach and Leiter, 2016), the factors causing stress often cannot be controlled. Stress can have positive effects such as increased engagement and performance (Liu *et al.*, 2019); however sustained job stress may lead to low work commitment and burnout, which is described as prolonged response to chronic interpersonal stressors on the job (Labrague *et al.*, 2018; Maslach and Leiter, 2016). Burnout is a syndrome characterized as a combination of high degree of both emotional exhaustion and depersonalization and low sense of personal accomplishment at work (Rotenstein *et al.*, 2018). Burnout can manifest itself in emotional exhaustion, feelings of cynicism and detachment from the job. Further manifestation may be a feeling of ineffectiveness as well as lack of accomplishment. (Maslach and Leiter, 2016). There is evidence that elevated workloads and role strain are associated with low organizational commitment among nurse leaders and a desire to leave their current employer (Labrague *et al.*, 2018; Wong and Laschinger, 2015). Additionally, VA Bogaert *et al.* (2014) found that one in six nurse managers experienced high or very high feelings of emotional exhaustion, and that role conflict and role meaningfulness were strong predictors of emotional exhaustion, work-related stress and turnover intentions among nursing unit managers.

Previous studies have shown that important sources of stress for nurse leaders include poor staffing, the expanding role of nurse leaders, complex work environments and heavy workloads (Remegio *et al.*, 2020; Labrague *et al.*, 2018). A recent systematic review (Penconek *et al.*, 2021) stated that nurse managers could face increased risks of both psychological and physical illness because of role stressors and inadequate coping strategies. In addition, nurse leaders were found to have difficulties in their work–life balance, partly due to a lack of work–life barriers (Kelly *et al.*, 2019). Strengthening factors that increase the work-related well-being of nurse managers could improve their health and reduce their job stress, both of which could have positive effects on retention. Some notable factors that enhance work-related well-being include recovery from work, the ability to “turn off” at the end of the workday, positive feedback and professional support from one’s leaders and colleagues (Herttuala *et al.*, 2020; Kelly *et al.*, 2019).

In spite of the need to improve recruitment and retention of nurse leaders, there are very few published studies on nurse leaders’ work-related well-being and factors affecting it. Those studies that are available have focused on stress and burnout as indicators of work-related well-being in this group. Here, this gap in the literature is addressed by examining nurse leaders’ work-related well-being and associated factors using a comprehensive approach that addresses nurse leaders’ experiences, resources, state of burnout and perceptions of their work conditions and professional capability.

## Method

### Aim

The study’s aim was to comprehensively describe nurse leaders’ experiences of work-related well-being and evaluate its associations with background variables, working conditions, work engagement, sense of coherence and burnout.

The research questions to be answered were:

RQ1. How do nurse leaders rate their overall experience of work-related well-being?

RQ2. How do nurse leaders perceive their working conditions, work engagement, sense of coherence and burnout?

RQ3. How are nurse leaders’ background variables, working conditions, work engagement, sense of coherence and burnout related to their overall experience of work-related well-being?

### Participants and recruitment

The participants of the study were nurse directors and nurse managers working in one of Finland’s largest organizations providing out- and inpatient social and health-care services to over 600,000 inhabitants. The organization has over 15,000 employees. The inclusion criteria were that participants had to work in units providing 24-h care. Nurse leaders (*N* = 350) from acute care facilities, mental health and substance abuse facilities, elderly services units, inpatient wards, emergency departments, rehabilitation and home hospital units were invited to take part in the study. Before data collection, a power analysis was conducted using RAOsoft (McDonald, 2014), indicating that a sample size of 153 was needed to achieve a confidence level of 90% and a margin of error of 5% based on a population size of 350. The information letter with a link to the questionnaire was first sent to a contact person of the health-care organization, who then sent it via email to all nurse leaders that met the abovementioned inclusion criteria.

### Data collection

Data was collected with an electronic survey between December 2015 and May 2016 using a questionnaire that included demographic questions and four internationally validated scales: the Utrecht Work Engagement Scale (UWES-9), QPS Nordic 34+, the shortened Sense of Coherence (SOC) scale and the Maslach Burnout Inventory (MBI). Before data collection, a pilot study involving 14 nurse leaders was conducted; no changes were made to the questionnaire based on the results of this pilot study.

The demographic questions provided information on the participants’ age, gender, current title, current division and workplace. Participants were also asked about their education, employment relationships and number of subordinates. In addition, participants assessed their leadership skills and well-being at work on a scale ranging from four (weakest) to ten (strongest).

Participants’ psychological, social and organizational working conditions were assessed using the QPS Nordic 34+ -instrument. Although it is not a health-related instrument itself, it may be used for the assessment of associations between work, health and productivity (Lindström *et al.*, 2000). The shortened version of this instrument was used because of the overlap of its themes with the questions of the other instruments used in this study. Seven subscales were used, namely, job demands (five items), work motivation (one item), control at work (four items), mastery of work (one item), leadership (two items), group work (two items) and health and well-being (one item). Participants answered the questions with five-point Likert scale (1 = “rarely or never” to 5 “very often or always”). The previously reported internal consistency of QPS Nordic 34+ has varied from acceptable to good (Tuominen, 2020; Lindström *et al.*, 2000) with Cronbach’s alpha values of 0.61–0.91 (Field, 2013).

The UWES-9 scale was used to measure participants’ work engagement. This instrument consists of three subscales (vigour, absorption and dedication) with three items each. Responses to these items were given using a seven-point Likert scale (1 = “never” to 7= “every day”). Previous reports indicate that this scale exhibits good internal consistency, with Cronbach’s alpha values of 0.70–0.94 (Ojala *et al.*, 2018; Seppälä *et al.*, 2009; Schaufeli and Bakker, 2004).

The SOC instrument was used to measure participants’ sense of coherence (Antonovsky, 1987). This instrument consists of 13 items divided over three subscales: comprehensibility (5 items), manageability (4 items) and meaningfulness (4 items). Responses to the items were given using a seven-point Likert scale (1= “always” to 7 = “never”). Previous studies (Gebrine *et al.*, 2019; Von Bonsdorff *et al.*, 2014) indicate that this instrument has good internal consistency, with Cronbach’s alpha values of 0.70–0.95 (Field, 2013).

Participants’ burnout levels were measured using three items of the MBI instrument, which was designed to measure three components of burnout syndrome: depersonalization, personal accomplishment and emotional exhaustion (Maslach *et al.*, 2015). In this study, one question relating to each component was presented to the participants, who responded using a seven-point Likert-scale (1 = “never” to 7 = “always”). Previous studies have shown that this instrument has good internal consistency (Matejic *et al.*, 2015; Maslach *et al.*, 2015; Kleijweg *et al.*, 2013), with Cronbach’s alpha values of 0.72–0.92 (Field, 2013).

### Analysis

The data was analysed using SPSS 27 for Windows. Descriptive statistics were used to characterize the participants’ demographic data. For further analysis, the participants’ age was recoded into four groups of approximately equal size (30–46, 47–51, 52–56 and 57–66 years), the number of employees was recoded into three groups (>20, 20–39 and 40–99) and the participants’ managerial level was recoded into two groups (manager and director). Finally, the participants’ work experience in healthcare was recoded into five groups (5–19, 20–24, 25–29, 30–35 and 36–42 years) and their experience in management was recoded into four groups (0–5, 6–10, 11–15 and 16–30 years).

The originally five-point scale of the QPS was recoded into three categories following the instructions of the user’s guide (Lindström *et al.*, 2000) in three dimensions. The new categories were 1 = seldom or never, 2 = occasionally and 3 = often or always in the dimensions of mastery of work and health and well-being. The new categories of work motivation were 1 = rather often, 2 = very often and 3 = always.

The relationships between variables were examined with Spearman’s correlations, with *r*-values above 0.5 and 0.3 being considered indicative of strong and intermediate correlations, respectively (Field, 2013). The non-normality of the data was confirmed using the Kolmogorov–Smirnov test. Non-parametric tests (the Mann–Whitney U test and the Kruskal–Wallis test) were used to investigate connections involving variables such as age or managerial level (Field, 2013).

A statistical significance threshold of *p* < 0.05 was applied. The internal consistency based on the calculated sum variables of QPS Nordic 34+, UWES-9 and SOC was evaluated by computing Cronbach’s alpha, with values > 0.7 being considered good and values of 0.6–0.7 being considered acceptable. (Field, 2013).

### Ethical considerations

Based on Finnish laws, no ethical approval was required for this study. However, the study followed the relevant ethical research guidelines (The World Medical Association, 2018), such as the guidelines of Finnish National Board on Research Integrity (Finnish Advisory Board for Research Integrity, 2012). Furthermore, the study was approved by the target organization. The participants participated voluntarily after receiving written information. (The World Medical Association, 2018). Data was collected anonymously and stored in a safe file accessible only to members of the research group (Nordic Nurses Federation, 2003). All scales used in this study were available on the internet.

## Findings

### Participants

A total of 155 nurse leaders participated in the study, as seen in Table 1, giving a response rate of 44%. Most of the participants were female (97%) and their mean age was 51 years (range 32–66 years). The most common degree-level qualification among the participants (39%) was a degree from a university of applied sciences, with smaller proportions holding a college degree (33%) or a university degree (28%). Their average experience in nursing was 24 years (range: 5–41 years), and they had ten years’ experience in nursing management on average (range: 1–30 years). Most of the participants worked as nurse managers (89%) under permanent contract (94%). Over a half of the participants (58%) were responsible for 20–39 subordinates. Most of the participants worked in acute care, rehabilitation or elderly services units (88%), followed by mental health and substance abuse facilities (9%) and family- and social services facilities (3%).

### Nurse leaders’ work-related well-being

Participants’ evaluations of their work-related well-being ranged between eight and ten (mean 8.12, SD 1.1). A minority (15.8%) gave a score of 10 for their work-related well-being, but the most commonly reported work-related well-being score was eight (43.9% of participants).

### Nurse leaders’ working conditions and work engagement

As shown in Table 2, group work had the highest reported scores (mean 4.31, SD 0.62) among the dimensions of working conditions, followed by leadership (mean 3.89, SD 1.07). The dimension with the lowest reported scores was job demands (mean 3.18, SD 0.52).

The dimension of work motivation was evaluated using a single question, which asked whether the participants found their work challenging. Most of the participants stated that their work rather often (42.7%) or very often or always (37.8%) challenging, but 13.4% stated that it was only occasionally challenging. The mastery of work dimension was also evaluated using a single item; most of the participants (62.7%) stated that they were often or always content with their ability to solve problems at work, but a minority (9.7%) said they were seldom or never content in this respect. Finally, 46.1% of the participants reported often or always experiencing stress, 37.0% said they occasionally experienced stress and 16.9% said they rarely or never experienced stress. A statistically significant correlation was found between participants’ experience of stress and their experience in nursing management (*r* = 0.231, *p* = 0.005), indicating longer experience to be associated with frequent experiences of stress.

As shown in Table 3, the work engagement dimensions with the highest score were dedication (mean 5.33, SD 0.80), followed by vigour (mean 5.15 SD 0.91) and absorption (mean 5.10, SD 0.91).

### Nurse leaders’ sense of coherence and levels of burnout

As seen in Table 4, among the dimensions of the Sense of Coherence scale, meaningfulness had the highest score (mean 6.18, SD 0.82), followed by manageability (mean 5.76, SD 0.68) and comprehensibility (mean 5.50, SD 0.80).

A majority (64.1%) of participants reported that they never experienced feelings of depersonalization, while 25.5% experienced such feelings seldom and 6.5% quite seldom. Minority of participants reported feelings of depersonalization occasionally (3,9%), none of the participants reported having these feelings quite often or always. Participants reported to have feelings of personal accomplishment often (38.8%), quite often (34.2%) and occasionally (21.7%). Minority reported feelings of personal accomplishment always (2.0%), quite seldom (1.3%) or seldom (2.0%); none of the participants chose the option never. A statistically significant difference (*p* = 0.017) was found between participants, who had these feelings quite often and occasionally, indicating the latter to have longer experience in nursing management. Furthermore, emotional exhaustion was reported seldom (38.6%), never (23.5%), quite seldom (15.1%) or occasionally (11.1%). Minority of the participants reported to have feelings of emotional exhaustion quite often (5.9%), often (3.9%) or always (1.3%). A statistically significant difference (*p* = 0.033) was found between the participants who had these feelings quite often and quite seldom, indicating the latter to have longer experience in nursing management.

### Factors related to nurse leaders’ work-related well-being

Correlations between participants’ work-related well-being and individual dimensions of the chosen scales are shown in Table 5. A weak (*r* = 0.192) but statistically significant (*p* = 0.044) correlation was found between participants’ leadership skills and their work-related well-being, indicating that nurse leaders with higher self-assessed skill levels experienced a higher level of work-related well-being. There was also a statistically significant correlation between participants’ work-related well-being and their current position (*r* = −0.177, *p* = 0.030): participants responsible for 20–39 employees had higher work-related well-being scores (mean 8.87, SD 0.75) than those responsible for fewer than 20 employees (mean 8.52, SD 0.66) or more than 39 employees (mean 8.62, SD 0.87) employees (*p* = 0.032).

No statistically significant correlations were found between participants’ work engagement and work-related well-being. There were statistically significant correlations between work-related well-being and all dimensions of working conditions, namely, job demands (*r* = −0.339, *p* = <0.001), control at work (*r* = 0.381, *p* = <0.001), leadership (*r* = 0.319, *p* = <0.001), group work (*r* = 0.366, *p* = <0.001), work motivation (*r* = −0.244, *p* = 0.002), mastery at work (*r* = 0.412, *p* = <0.001) and health and well-being (*r* = −0.579, *p* = <0.001). Participants who found their work to be challenging rather often reported higher levels of work-related well-being than other groups (*p* = 0.011).

Participants’ work-related well-being also correlated with two dimensions of sense of coherence, namely, meaningfulness (*r* = 0.192, *p* = 0.018) and manageability (*r* = 0.248, *p* = 0.003). In addition, a statistically significant correlation was found between work-related well-being and participants’ emotional exhaustion (*r* = −0.484, *p* = <0.001). Participants, who never experienced emotional exhaustion had statistically significantly higher level of work-related well-being (mean rank 91.53) than participants who experienced emotional exhaustion quite often (mean rank 24.40, *p* = 0.003), quite seldom (mean rank 54.96, *p* = 0.005), occasionally (mean rank 53.87, *p* = 0.021) or often (mean rank 9.00, *p* = 0.048).

## Discussion

### Discussion about study results

This study provided new insights into nurse leaders’ work-related well-being by adopting a comprehensive approach reflecting the multifaceted nature of work-related well-being. This multifaceted nature has been recognized previously (Kelly *et al.*, 2019; Trinchero *et al.*, 2014; Brunetto *et al.*, 2013; Buffet *et al.*, 2013); assessing work-related well-being from an overly narrow perspective (e.g. one focusing only on stress and burnout) results in an incomplete understanding of the phenomenon. Our results show that work-related well-being is affected by both organizational factors such as working conditions and personal factors such as sense of coherence. These findings provide a deeper understanding of nurse leaders’ work-related well-being and could be used to inform and guide efforts to increase work-related well-being by targeting beneficial factors.

Almost half of the participants experienced stress often or always. This is notable because stress and burnout are growing problems among nurse leaders (Remegio *et al.*, 2020; Saifman and Sherman, 2019; Djukic *et al.*, 2017), possibly owing to the expanding scope of nurse leaders’ roles in health-care organizations (Nurmeksela *et al.*, 2020). Our results also show that higher levels of experienced stress were associated with lower levels of work-related well-being. However, the study participants had high levels of perceived work-related well-being. This shows that work-related well-being should not be evaluated on the basis of just one factor. Nurse leaders may have resources such as leadership skills or a strong sense of coherence that prevent experiences of stress from adversely affecting their work-related well-being. This is further supported by our results, that revealed that even when longer experience in nursing management was associated with more frequent experiences of stress, it also strengthened participants’ feelings of personal accomplishment and decreased the emotional exhaustion. It is well known that distress, the negative response to stressors, is negative and may have adverse health outcomes. It is important to note that there is also good stress, known as eustress and this is associated with positive outcomes. It is also stated that not all stress nurses experience, is negative, but can for example maintain the ability to respond to pressure in their work (Liu *et al.*, 2019; Adriaenssens *et al.*, 2017; Simmons and Nelson, 2001). Future research should thus pay more attention to factors that empower nurse leaders by strengthening their work-related well-being and how the eustress manifests itself in nurse leaders.

No correlation between work-related well-being and work engagement was observed in this work, even though previous studies have linked work engagement to work-related well-being in medical laboratory settings (Narainsamy, 2013). Also, Remegio *et al.* (2020) found that work engagement was related to nurse leaders’ compassion satisfaction, which is defined as the pleasure a person derives from their ability to do work well. Although compassion satisfaction can be seen as a concept that is closely related to work-related well-being, it is to notice that the meaning is slightly different. Therefore, the relationship between work-related well-being and work engagement should be studied closely in the future. On the other hand, working conditions seemed to significantly affect work-related well-being. The results presented here suggest that work-related well-being is positively related to one’s level of control at work, the leadership skills of one’s superiors and the level of teamwork in the workplace. This is consistent with previous reports (Herttuala *et al.*, 2020; Kelly *et al.*, 2019). Organizations wishing to increase nurse leaders’ work-related well-being and commitment should therefore try to improve their working conditions by giving them greater autonomy and by promoting shared and participatory leadership (Kanninen *et al.*, 2021). In addition, organizations should maintain and improve the leadership skills of nurse leaders by giving them access to education and mentoring programmes to enable and support their professional growth.

There was a negative correlation between current position and work-related well-being. This could be due to the responsibilities associated with different positions: employees at higher levels of the managerial hierarchy have broader responsibilities and their decisions affect greater numbers of employees. Supporting this hypothesis, participants who managed 20–39 employees reported the highest levels of work-related well-being. Self-assessed leadership skills were also associated with higher levels of work-related well-being. In future, the relationship between managerial level and work-related well-being could be investigated by studying the leadership skills, empowerment and experienced manageability of nurse leaders working at higher levels of management.

Other factors positively associated with work-related well-being among nurse leaders were contentment with one’s own problem-solving abilities, finding work to be frequently challenging, and a high sense of coherence. This indicates that work-related challenges and meaningfulness of work are positive factors for nurse leaders who are confident in their leadership skills and abilities. In keeping with this conclusion, we also observed a positive correlation between personal accomplishment and work-related well-being. Consequently, it would be beneficial to study and enhance factors that empower nurse leaders. The need for such studies is strengthened by the finding that work-related well-being correlated negatively with depersonalization and emotional exhaustion.

Nurse leaders also need training to support their work-related well-being, and health-care organizations need to learn how retain new nurse managers (Djukic *et al.*, 2017). Penconek *et al.* (2021) and Melnyk *et al.* (2020) acknowledged in their systematic reviews that nurse leaders would benefit from interventions that reduce stress and increase work-related well-being. In their systematic review, Häggman-Laitila and Romppanen (2018) found that stress management interventions were the most frequently studied and most successful interventions for nurse leaders. However, interventions and programs often seem to focus on individuals and to be problem focused. (Häggman-Laitila and Romppanen, 2018). Because of the characteristics of the work of the nurse leaders, future interventions should be developed and focused more broadly on the collaboration relationships between leaders and nursing staff. Moreover, organizations should implement well-being strategies that incorporate multiple actions to strengthen individual skills and teamwork while also improving working conditions and stress management rather than just focusing on a single aspect of work-related well-being. The results presented here clearly indicate that a proper evaluation of nurse leaders’ work-related well-being requires the consideration of multiple factors as well as specific training and mentoring at the leadership level where they currently work. Such support could help ensure the future well-being and empowerment of leaders in the health-care sector.

### Recommendations

Working conditions, and teamwork in particular, strongly affect nurse leaders’ work-related well-being. Therefore, collaboration between nurse leaders and shared leadership practices should be supported and enabled at all levels of organization. Leadership is another important aspect of working conditions, so organizations should ensure high-quality leadership practices by offering education, various interventions and mentoring at all managerial levels. Furthermore, the organizations should pay attention to the difference between distress and eustress and employ the positive aspects of eustress in development and implementation of educational interventions for nurse leaders. Also, the organizations should offer leaders education regarding their work-related well-being and the factors affecting it and involve them to enhance it.

Nursing leadership is seen as challenging work and nurse leaders often experience stress, especially with limited experience in nursing management, which reduces their work-related well-being. To decrease the nurse leaders’ experienced stress, the organizations should implement policies (e.g. flexible working hours or possibility of remote work) and practices that support the balance between nurse leaders’ work and private life. In addition, organizations should enable and support nurse leaders’ professional development and ensure them appropriate resources to meet the demands of their work. The nurse leaders consider their work meaningful in spite of its demanding nature, which is indicated by their rather strong sense of coherence. Organizations should enhance the nurse leaders’ experiences of the meaningfulness of their work, by offering them tasks that challenge their competences in a positive way as well as feedback about their performance.

Organizations should also ensure regular development discussions to the leaders, including a comprehensive evaluation regarding their work-related well-being and how to strengthen it. Organizations should also ensure that they appreciate in their strategy the work-related well-being of their leaders as high as of their nursing staffs. At the same time, it is paramount to realize that these two groups need very different support for their work-related well-being from their organizations.

### Strengths and limitations

All of the study’s participants were from the same region of Finland, which could be seen as a limitation. Additionally, the vast majority of the participants were nurse managers; it would have been interesting if more directors of nursing had participated. The evaluations of work-related well-being and related factors were based on self-reports, so the results obtained should be interpreted with caution. A final limiting factor is that the concept of work-related well-being is subjective, meaning different things to different people. The strengths of this study are an adequate sample size based on power analysis conducted prior to data collection, a good response rate of 44% and acceptable to good (*α* ≥ 0.64) internal consistence of the instruments that are in line with previously reported levels of Cronbach’s alphas. The chosen instruments are widely used in the international research (Tuominen, 2020; Gebrine *et al.*, 2019; Ojala *et al.*, 2018; Matejic *et al.*, 2015; Maslach *et al.*, 2015; Von Bonsdorff *et al.*, 2014; Kleijweg *et al.*, 2013; Seppälä *et al.*, 2009; Schaufeli and Bakker, 2004; Lindström *et al.*, 2000) related to the current area of interest. Thus, it was estimated that the instruments are measuring relevant themes, indicating a strong content validity. Previous studies have also demonstrated the cultural adaptability of the instruments (Tuominen, 2020; Ojala *et al.*, 2018; Seppälä *et al.*, 2009). It is to notice, however, that QPS Nordic 34+ is not a health-related instrument itself, but it may be used in research by assessment of associations between work, health and productivity (Lindström *et al.*, 2000).

## Conclusion

This study indicated that narrow inspections of work-related well-being focusing mainly on issues such as stress and burnout cannot reveal all aspects affecting nurse leaders’ work-related well-being: the participants had high levels of perceived work-related well-being even though almost half of them reported experiencing stress often or always. Our study suggests that nurse leaders may have resources (such as leadership skills or a high sense of coherence) that prevent the experienced stress from negatively influencing their work-related well-being. Working conditions also seemed to significantly affect work-related well-being; it correlated positively with the participants’ level of control at work, the reported leadership skills of their superiors and the level of teamwork in the workplace. Work-related well-being was also positively associated with contentment with one’s own problem-solving abilities and finding work to be frequently challenging. These findings are important given the concerns about the attractiveness of nurse leadership as a profession. Future research should pay more attention to empowering interventions targeting personal factors and working conditions to improve the work-related well-being of nurse leaders.

## Figures and Tables

**Table 1.** Participants' demographics (*n* = 155)

	f	(%)
*Gender (n = 152)*		
Male	4	2.6
Female	148	97.4
*Age in years (n = 151)*		
30–46	36	23.8
47–51	47	31.1
52–56	32	21.2
57–66	36	23.8
*Level of education (n = 154)*		
College	51	33.1
University of applied sciences	60	39.0
University degree	43	27.9
*Managerial level (n = 153)*		
Manager	136	88.9
Director	17	11.1
Workplace (*n* = 155)	136	87.7
*Acute care, rehabilitation or elderly services units*		
Mental health and substance abuse facilities	14	9.0
Family- and social services facilities	4	2.6
*Number of subordinates (n = 137)*		
>20	45	32.8
20–39	79	57.7
40–99	13	9.5
*Experience in healthcare in years (n = 151)*		
5–19	33	21.9
20–24	36	23.8
25–29	32	21.2
30–35	34	22.5
36–41	16	10.6
*Managerial experience in years (n = 145)*		
0–5	40	27.6
6–10	61	42.1
11–15	18	12.4
16–30	26	17.9

**Table 2.** Participants’ scores for different dimensions of working conditions (*n* = 155)

Dimension	No. of items	n	Min	Max	Mean	SD	α
Job demands	5	151	2.00	4.80	3.18	0.52	0.64
Control at work	4	148	1.00	5.00	3.53	0.73	0.76
Leadership	2	152	1.00	5.00	3.89	1.07	0.90
Group work	2	154	1.00	5.00	4.31	0.62	0.73

**Note:** *α* = Cronbach’s alpha

**Table 3.** Participants’ scores for different dimensions of work engagement (*n* = 155)

Dimension	No. of items	*n*	Min	Max	Mean	SD	*α*
Vigour	3	151	2	6	5.15	0.91	0.90
Absorption	3	150	2	6	5.10	0.87	0.73
Dedication	3	152	3	6	5.33	0.80	0.88

**Note:** *α* = Cronbach’s alpha

**Table 4.** Participants’ scores for different dimensions of sense of coherence (*n* = 155)

Dimension of SoC	No. of items	*n*	Min	Max	Mean	SD	*α*
Comprehensibility	5	152	2	7	5.50	0.80	0.78
Manageability	4	145	3	7	5.76	0.68	0.67
Meaningfulness	4	152	4	7	6.18	0.82	0.67

**Note:** *α* = Cronbach’s alpha

**Table 5.** Correlations between work-related well-being and factors studied in this work

	Work-related well-being	
Associated factors	*r*	*p*
Managerial skills	0.192	0.044
Current position	−0.177	0.030
Job demands	−0.339	<0.001
Control at work	0.381	<0.001
Leadership	0.319	<0.001
Group work	0.366	<0.001
Work motivation	−0.244	0.002
Mastery at work	0.412	<0.001
Health and well-being	−0.579	<0.001
Meaningfulness	0.192	0.018
Manageability	0.248	0.003
Depersonalization	−0.192	0.018
Personal accomplishment	0.177	0.030
Emotional exhaustion	−0.545	<0.001
